# Nanotechnology in Clothing and Fabrics

**DOI:** 10.3390/nano12010067

**Published:** 2021-12-28

**Authors:** Muralidharan Paramsothy

**Affiliations:** NanoWorld Innovations (NWI), 1 Jalan Mawar, Singapore 368931, Singapore; mpsothy@yahoo.co.uk

Making clothing and fabric with nanoparticles or nanofibers allows the improvement of fabric properties without a significant increase in weight, thickness, or stiffness. For example, incorporating nano-whiskers into fabric used to make pants produces a lightweight water- and stain-repellent material. Selected companies involved in this fast-developing arena include Nano-Tex, Aspen Aerogel, BASF, and Nano-Horizons. In line with this theme, four representative articles [1,2,3,4] have been published in this Special Issue:

As shown by Kan et al. [1], the preparation of poly(styrene-methyl methacrylate-acrylic acid) deposited carbon fabrics (or Poly(St-MMA-AA)/PCFs) with desirable vivid structural colours transforming remarkably from salmon to medium purple (by controlling the Poly(St-MMA-AA) size) was feasible. The concentric shape of Poly(St-MMA-AA), with Poly(MMA-AA) shell and PS core, as well as the chemical composition of the Poly(St-MMA-AA) copolymer, were investigated. The structural colours in the visible spectrum could be predicted and verified by calculation of the sizes of as-synthesised Poly(St-MMA-AA) nanoparticles according to Bragg’s and Snell’s laws. The colour difference of Poly(St-MMA-AA)/PCFs with adjacent sized Poly(St-MMA-AA) nanoparticles was measured. The obvious changes of surface resistivity among carbon fabrics, Poly(St-MMA-AA)/PCFs under dry and wet conditions were investigated. The deposition of Poly(St-MMA-AA) photonic crystal films increased the resistivity of the carbon fabrics, and then decreased after dipping treatment. The proposed method provided Poly(St-MMA-AA)/PCFs the potential applications in smart displays for traffic warning signals, taking into account the challenge of large-scale fabrication of Poly(St-MMA-AA)/PCFs.

Blosi et al. [2] obtained multifunctional luminescent textiles by applying for the first time new iridium supported silica (Ir@SiO_2_) compounds in the form of coating. Treated textiles displayed very promising self-marking properties together with antibacterial activity. The strong interaction between Ir complexes and silica nanoparticles enabled easy detection under UV light, both when used as coating for textiles and after release in the environment. The homogeneous distribution of such luminescent coating can be usefully exploited as a wear diagnostic tool. In addition, the antibacterial testing showed interesting light-induced antimicrobial properties against both Gram(+) (Deinococcus radiodurans) and Gram(−) (Escherichia coli) bacteria systems, probably due to production of reactive oxygen species (ROS). For useful application, a preliminary human dermal exposure assessment candidate such as Ir@SiO_2_ coating (as a safety by design solution) can be easily tracked for the whole life cycle, i.e., from production to release of nanoparticles.

In work by Zhang et al. [3], the enhanced photocatalytic activity of a composite photocatalyst composed of polyethylene terephthalate (PET) filaments loaded with Ag-N co-doped TiO_2_ nanoparticles sensitized with water-insoluble disperse blue 183 dye (which was formed in a facile one-step hydrothermal process under visible light irradiation) was achieved. The synergetic mechanism of both metal-nonmetal co-doping and dye sensitization on the photocatalytic activities of TiO_2_ nanoparticles under visible light irradiation was investigated. The superior photocatalytic activity of the dye-sensitized PET-Ag-N-TiO_2_ composite photocatalyst was primarily due to the quick separation of photo-generated electron-hole pairs and efficient interfacial charge transfer. The results of photo-degradation of methyl orange (MO) dye solution (with trapping experiments as well as ESR measurements) showed that the holes, in comparison with •OH and •O_2−_ radicals, were the main reactive radical species for the PET filaments coated with dye-sensitized Ag-N co-doped TiO_2_. PET substrate and water insoluble disperse blue dye greatly contributed to the superior photocatalytic activity of the dye-sensitized Ag-N co-doped TiO_2_ coated PET filaments. This not only generated more holes, but also inhibited the recombination of electron-hole pairs by the transfer of electrons to the dyed PET substrate and Ag nanoparticles. In comparison to the PET filaments coated with TiO_2_ nanoparticles, the PET filaments coated with Ag-N co-doped and dye-sensitized TiO_2_ nanoparticles exhibited greatly enhanced light absorption capacity, efficient separation of electron-hole pairs, and substantial photocatalytic activity in degradation of MO dye under visible-light irradiation. The resultant photocatalytic composite filaments were capable of repeatedly photo-degrading MO dye without losing initial photocatalytic activity significantly. The proposed photocatalytic composite structure can be taken as a novel approach to design textile materials based composite photocatalysts for the photo-degradation of organic pollutants.

As reported by Lin et al. [4], highly sensitive electrocardiogram (ECG) electrodes from electrically conductive films containing nanoscale silicate platelets/polyoxyethylene-segmented polyimide/Ag nanoparticles (NSP/POE-imide/AgNPs) nanohybrids were fabricated. The use of POE-imide as an organic dispersant and NSP as an inorganic dispersant allowed AgNPs to be well-dispersed in a DMF/water co-solvent system. Through a facile coating and sintering process, hybrid polymer-mediated films were made that exhibited low surface resistances of up to 10^−4^ W/sq. Furthermore, the surface resistance of the hybrid films appeared to decrease due to the interconnected network formed by the AgNPs. The results indicated that these films are promising as nano-conductors in electronic devices. Silver-based electrodes show demonstrated potential not only in biosensor ECG systems for monitoring cardiac activity, but also in functional biological applications. However, exploring the large-scale production of these films is dependent on commercial viability and uptake. The use of dry electrodes for ECG devices is an exciting application for wearable technology featuring nanohybrid films.
